# Conformational Transitions of Polymer Chains in Solutions Characterized by Fluorescence Resonance Energy Transfer

**DOI:** 10.3390/polym10091007

**Published:** 2018-09-10

**Authors:** Linlin Qin, Linling Li, Ye Sha, Ziyu Wang, Dongshan Zhou, Wei Chen, Gi Xue

**Affiliations:** 1 Department of Polymer Science and Engineering, School of Chemistry and Chemical Engineering, Nanjing University, Nanjing 210093, China; qinlinlinnju@163.com (L.Q.); llli@nju.edu.cn (L.L.); mg1224056@smail.nju.edu.cn (Y.S.); dzhou@nju.edu.cn (D.Z.); weichen@nju.edu.cn (W.C.); 2 State Key Laboratory of Pollution Control and Resource Reuse, School of the Environment, Nanjing University, Nanjing 210046, China; wangziyu17@126.com; 3 Guangdong Provincial Key Laboratory of Nano-Micro Materials Research, School of Chemical Biology and Biotechnology, Peking University Shenzhen Graduate School, Shenzhen 518055, China

**Keywords:** polymer solution, conformational transition, critical overlap concentration, fluorescence, viscosity

## Abstract

The critical overlap concentration C* is an important concept in polymer solutions and is defined as the boundary between dilute and semidilute regimes. In this study, the chain conformational changes of polystyrene (PS) with both high (*M*_n_ = 200,000 Da) and low (*M*_n_ = 13,000 Da) molecular weights in cis-decalin were compared by intrachain fluorescence resonance energy transfer (FRET). The random labeling of donor and acceptor chromophores strategy was employed for long PS chains, whereas chain-end labeling was used for short PS chains. By monitoring the spectroscopic intensity ratio between acceptor and donor, the concentration dependence on chain conformation from dilute to semidilute solutions was determined. Both long and short chains exhibit a conformational transition concentration, above which the polymer chains begin to collapse with concentration significantly. Interestingly, for randomly labeled polymer long chains, such concentration is consistent with C* determined from the viscosity result, below which only slight conformational change of polymer chain takes place. However, for the chain-end labeled short chain, the conformational transition concentration takes place earlier than C*, below which no significant polymer conformation change is observed.

## 1. Introduction

Given its theoretical and practical importance, it is of great value to understand the relationship between polymer conformation and concentration. On the basis of scaling theory [1], polymer solutions can be divided into three different regions: the dilute, semidilute, and concentrated solutions. There is a fundamental distinction between the dilute regime, where the polymer coils are separated from each other, and the semidilute regime, where the polymer coils overlap and interpenetrate. A threshold termed critical overlap concentration, C*, exists from the dilute to semidilute solution, at which the polymer coils begin to contact each other [2,3,4,5,6,7,8].

The conformation of polymer has been provided by many experimental methods. Among them, the methods of scattering, including light scattering [7,9,10], neutron scattering [11,12,13,14,15], and X-ray scattering [16,17], are the most widely employed techniques. However, de Gennes noted that it was only proper to explore at the distance of 2–50 nm by small-angle neutron scattering [11]. The study of polymer conformation at a smaller dimension scale is invalid by these scattering methods.

Fluorescence spectroscopy has been considered as a common method to characterize polymer solutions [18,19,20,21]. By using an excimer fluorescence experiment, Qian proposed the conformational transition point *Cs* at which polymer coils began to contract. *Cs* was demonstrated to be two orders of magnitude lower than C* [22]. Contrary to this, Torkelson claimed that in a polystyrene (PS) system between dilute and semidilute solutions, there was no abrupt transition found by using excimer fluorescence spectroscopy [23]. In addition to the excimer fluorescence technique, fluorescence resonance energy transfer (FRET) has been employed to study polymer conformation transition [24,25,26,27]. According to the theory of Förster, FRET efficiency is inversely proportional to the sixth power of the distance between donor and acceptor, and the effective distance range is 0.5–10 nm [28]. Correspondingly, the ratio of intensities of the fluorescence peaks of acceptor to donor in the fluorescence spectrum can be used as the “spectroscopic ruler” to measure the distance between the donor and acceptor [29]. After grafting fluorescent acceptors and donors onto polymer chains, the conformation and interaction of polymer chains can be simplified by statistically measuring the distance between the acceptors and donors [30,31]. FRET provides a robust method to investigate the conformational evolution for polymers with low molecular weights, which is far less considered [32].

In this work, FRET was employed to study the conformational transition of polymer chains from dilute to semidilute solutions with two different labeling strategies. A random dye-labeling strategy was utilized for PS with *M*_n_ = 200,000 Da, while a site-specific terminal labeling strategy was employed for PS with *M*_n_ = 13,000 Da. According to the ratio of anthracene and carbazole intensities (*I_A_*/*I_C_*), the conformation of a single chain could be detected. The conformational transition result of fluorescence spectroscopy was then compared with the C* value determined from a viscosity experiment.

## 2. Materials and Methods

### 2.1. Materials

Polystyrene (PS, *M*_n_ = 2.05 × 10^5^, PDI = 1.06; *M*_n_ = 1.25 × 10^3^, PDI = 1.10) obtained from Polymer Source Inc. Styrene (from Aladdin, Shanghai, China) was treated by neutral alumina and distilled before experiments. CuBr was washed with acetic acid and stored in N_2_. 9-Anthracenemethanol, carbazole, chloromethyl methyl ether, SnCl_4_, 4-dimethylaminopyridine (DMAP), dicyclohexylcarbodiimide (DCC), 4,4′-dinonyl-2,2′-bipyridyl(dnbipy), NaN_3_, *N*,*N*,*N*,*N″*,*N″*-pentamethyldiethylenetriamine (PMEDTA), and 2-bromopropionic acid were obtained from Aladdin Reagents, TCI Chemicals (Shanghai, China), and J&K Chemicals (Shanghai, China). Solvent DMF, CCl_4_, methanol, and THF were purchased from Tansole Reagent (Shanghai, China).

### 2.2. Samples Synthesis

**Scheme 1:**
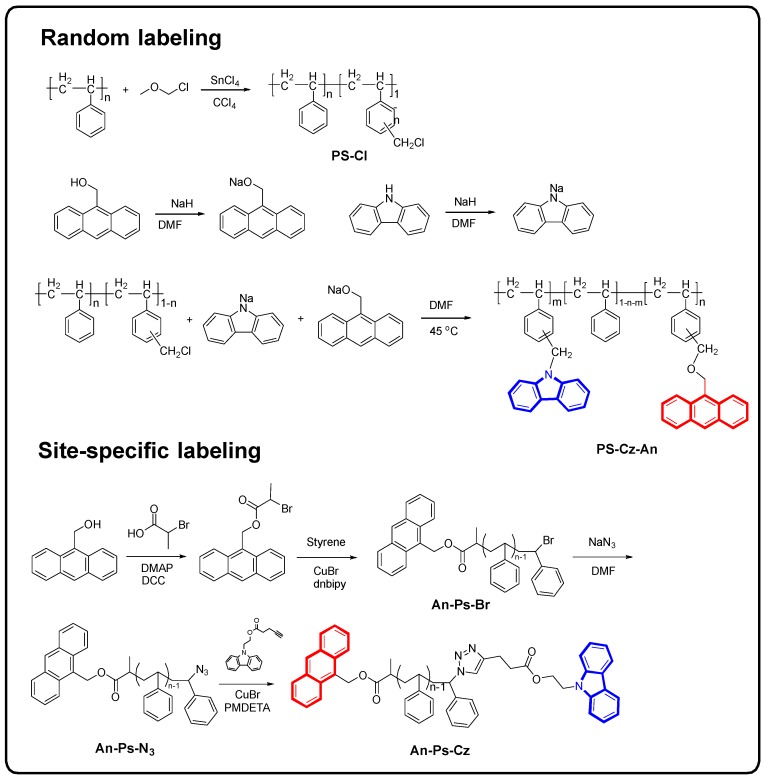
Synthetic routes of polystyrene (PS) labeled with fluorescent donor and acceptor randomly on chains and specifically in the ends.

**PS-Cz-An** PS samples labeled randomly with carbazolyl and anthryl (PS-Cz-An) were prepared via a modified strategy based on Morawetz’s work [33], as shown in Scheme 1. PS (5 g) was added into 100 mL CCl_4_ and dissolved for 3 h under argon. After complete dissolution, 5 mL chloromethyl methyl ether and 5 mL SnCl_4_ were injected into the solution at room temperature and fully stirred for 5 min. To quench the reaction, the whole solution was poured into methanol for precipitation to get PS-Cl. The labeling ratio of methylene chloride was determined from ^1^H NMR. Under the protection of Ar, carbazole (0.32 g) and 9-anthracenemethanol (0.4 g) were dissolved in 30 mL dry DMF. NaH (0.31 g) was added dropwise into the solution within 20 min. The reaction lasted 6 h in a 45 °C oil bath. Under the protection of argon, PS-Cl (2 g) was dissolved in DMF. A 1.2-mL solution of sodium carbazole and sodium anthrol was injected into the PS-Cl solution. When the reaction time was reached, the whole solution was poured into the methanol for precipitation. The content of fluorescent donor and acceptor was measured by UV spectrophotometer and compared with a model compound.

**An-PS-Cz** Dye-labeled PS samples (An-PS_13k_-Cz) were synthesized in our laboratory through atom transfer radical polymerization (ATRP) and click reaction [31]. All experiments were conducted under anhydrous and anaerobic conditions. Under argon protection, 9-anthracenemethanol (2.5 g, 1.2 mmol), 2-bromopropionic acid (2.203 g, 1.32 mmol), and DMAP (0.1466 g, 0.12 mmol) were dissolved in 30 mL CH_2_Cl_2_ and stirred for 30 min at 0 °C. DCC (1.981 g, 1.2 mmol) was dissolved in 15 mL CH_2_Cl_2_ and the mixture was added into the above solution within 15 min. The reaction lasted for 6 h. The product was filtered by funnel and the filtrate was washed with NaHCO_3_ solution, deionized water, saturated brines, and dried with anhydrous Na_2_SO_4_. According to the designed molecular weight and the molar ratio of initiator, catalyst:ligand = 1:1:2, styrene (10 g, 0.1 mol), initiator (0.1662 g, 0.48 mmol), CuBr (0.0687 g, 0.48 mmol), and dibnpy (0.3924 g, 0.96 mmol) were added into the flask to react, then sealed and placed in an oil bath to react at 110 °C. The resulted copper salts were removed by a neutral alumina column and precipitated into methanol. The product of An-PS_13k_-Br was obtained. An-PS-Br (5.2 g, 0.04 mmol) was dissolved in 30 mL DMF solution and NaN_3_ (0.1271 g, 0.2 mmol) was then added. The reaction lasted for 2 days at 60 °C under the protection of argon. After the reaction, polymer was precipitated into methanol for purification and the product of An-PS-N_3_ was obtained. In order to get An-PS_13k_-Cz, An-PS_13k_-N_3_ (4.5 g, 0.34 mmol) and carbazolylalkyne (0.1478 g, 0.51 mmol) were firstly dissolved in 30 mL DMF, then CuBr (0.0243 g, 0.17 mmol) and PMEDTA (0.293 g, 0.17 mmol) were added. The entire reaction lasted for 2 days in a glove box. Then, polymer was precipitated into methanol and evaporated and conserved in Ar. An-PS_9.7k_-Cz was obtained by a similar synthetic path. All the polymer samples are shown in Table 1. 

### 2.3. Measurement

The average molecular weights of PS and An-PS-Cz were measured with the PL-GPC 120 system, taking THF as the moving phase at the elution rate of 1 mL/min. The molecular weight was calibrated by narrow dispersed PS. The labeling ratio of fluorescent groups was determined by a MAPADA UV-1800 spectrophotometer (MAPADA, Shanghai, China). The fluorescence spectra were recorded on a PTI (Photon Technology International) spectrofluorometer with an excitation wavelength of 294 nm. The NMR spectra were recorded on a Bruker DRX-400 spectrometer (Bruker, Karlsruhe, Germany) and CDCl_3_ was used as the solvent with TMS as internal calibration.

## 3. Results and Discussion

The structure of polymer coils in solutions shows a dependence on concentration, and there usually exists a conformational transition as the concentration increases past the C*. Such conformational transition from a dilute to semidilute solution is a process involving both interchain and intrachain conformations. Based on the intuitive scenario of de Gennes’ theory, most of the previous studies focused on the characterization of critical overlap concentration by the interchain conformation. However, few of them explained the synergetic intrachain conformation transition. In this work, the fluorescent pairs were attached onto a single polymer chain, which allowed us to characterize the associated intrachain conformations by FRET technology.

**Figure 1:**
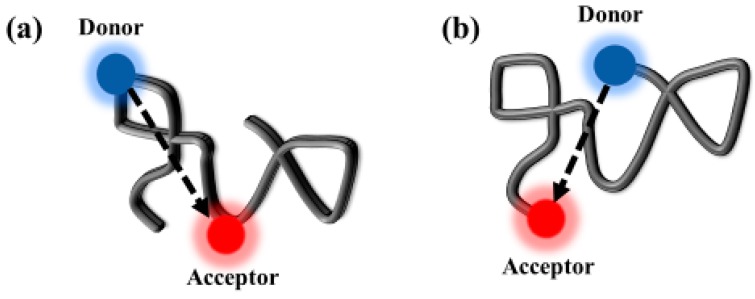
Schematic graphs for two types of intrachain fluorescence resonance energy transfer (FRET) methods classified by the varied fluorescent chromophores labeling strategies: (**a**) random labeling, where the donor and acceptor are randomly attached onto one single polymer chain; and (**b**) chain-end labeling, where the donor and acceptor are precisely fixed at chain terminals.

FRET has been proven as a spectroscopic ruler for characterizing the distance between fluorescent donor and acceptor [29]. Usually, by controlling the labeling strategies of such chromophores, different kinds of physical properties sensitive to distance can be explored [3,34,35,36,37,38,39]. For linear polymer chains, there exists two types of intrachain labeling strategies: random labeling, where the two chromophores are attached randomly onto one single chain together, and chain-end labeling, where the fluorescent acceptor and donor are fixed at the ends of polymer chains with the aid of site-specific ATRP and click chemistry (seen in Figure 1b) [31,40,41]. The latter chain structure shows high reliability in the calculation of polymer chain end-to-end distance. According to the theory formulated by Fӧrster, the FRET efficiency (*E*) and donor–acceptor distance (*r*) obey the equation *E* = (1 + (*r*/*R*_0_)^6^)^−1^, where *R*_0_ is the characteristic distance of the selected FRET pairs. For the widely used FRET pair of carbazole (**Cz**) and anthracene (**An**), *R*_0_ is about 3 nm [42]. This means that the FRET method is available when the **Cz**–**An** distance is in the range of 1–5 nm and is extremely sensitive when the distance is around 3 nm. The strategy of random labeling does not have a clear requirement for molecular weight as long as the polymer chain conformation allows the distance between the donor and acceptor to be within the fluorescence effective range. One drawback of FRET experiments conducted on end-labeled monodisperse polymer chains is that the polymer chain cannot be too long or else the ends will be too far to undergo FRET, and a weak or even no signal would be obtained. Thus, FRET experiments on end-labeled monodisperse chains can only be conducted on short chains.

Owing to the low volatility and tunable solvent quality with temperature, cis-decalin (DHN) was used as the solvent in this study. For dye-labeled PS chains in DHN solution, the FRET signals may come from two counterparts: intrachain FRET and interchain FRET. The existence of the latter would complicate the relationship between the FRET signal and the size of polymer coils. In order to eliminate the influence of interchain FRET, blank PS without dyes was added to shield interchain FRET response. Moreover, to completely inhibit the reabsorption effect, the amount of dye-labeled polymer in all solutions was fixed at a constant value, and the concentrations were tuned by adding varied quantities of blank polymers. When the PS samples labeling **Cz** and **An** were excited at the wavelength of 294 nm, the energy transfer from **Cz** to **An** would occur, accompanied by a decrease of *I_C_* (**Cz** intensity) at wavelength 362 nm and an increase of *I_A_* (An intensity) at wavelength 414 nm. The ratio of *I_A_* to *I_C_* reflects the distance between **Cz** and **An**, which implies the coil size of polymer chains.

**Figure 2:**
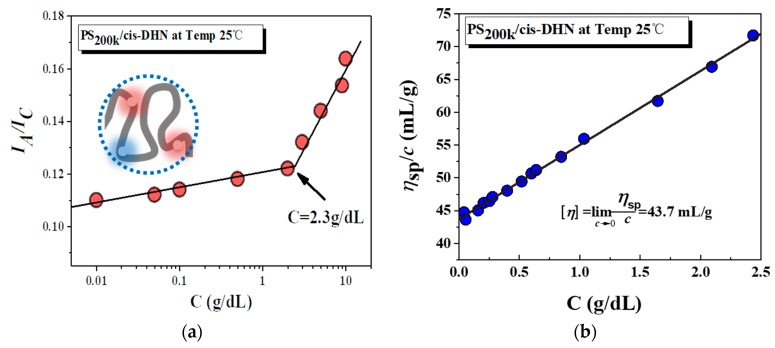
(**a**) The *I_A_*/*I_C_* value as a function of concentration for PS_200k_ doped by PS-An-Cz in cis-decalin (DHN) at 25 °C. (**b**) The reduced viscosity *η_sp_*/*c* as a function of concentration for PS_200k_ in DHN at 25 °C.

Figure 2a shows the fluorescence data of PS_200K_ with acceptor and donor randomly labeled onto the chain. According to the dependence of *I_A_*/*I_C_* with polymer concentration, it displays a break point and two regions are identified: (1) a slight increase of *I_A_*/*I_C_* value with the increase of concentration in dilute solutions before the break; and (2) a sharp raise of *I_A_*/*I_C_* value with the increase of concentration after the break. The break point from dilute to semidilute solution for the PS_200K_/DHN system is 2.3 g/dL. Based on the scaling theory [1], it is considered that polymer coils are isolated and its physical properties are dominated by individual polymer coils in a dilute solution, but it is not clear when it will happen. It is clear that slight increases of *I_A_*/*I_C_* exist in dilute solution for PS_200k_, which means there is still weak interaction between the long polymer chains. Because of this weak interaction, a slight collapse of the individual chain occurs before C*. Nishinara et al. [43] studied the chain conformation by the ratio of excimer to monomer fluorescence intensity, *I_E_*/*I_M_*, and proved that *I_E_*/*I_M_* has a linear relationship with the increase of concentration in a dilute solution of PS (*M*_v_ = 2,000,000 Da). As the concentration becomes higher, the proximity of polymer chains gradually becomes closer. This means that polymer chains no longer act individually and can be strongly affected by adjacent chains. When a certain concentration is reached, polymer coils become densely packed and shrink, leading to a sharp increase of *I_A_*/*I_C_* values.

As is well known, the reciprocal of the intrinsic viscosity [*η*] could be used to estimate the critical overlap concentration of polymer solutions [44]. In order to confirm that the results of fluorescence measurements match the conformational transition of PS_200k_ in solution, this was further proved by the viscosity experiment. Figure 2b shows the linear relationship between reduced viscosity and concentration of PS_200K_/DHN solutions, and the intrinsic viscosity can be calculated through the intercept ([*η*] = 43.7 mL/g). As a result, the critical overlap concentration of the viscosity experiment is 2.3 g/dL, which is consistent with the fluorescent data. The combination of such experimental results show that the intrachain FRET method can be used to describe the critical overlap concentration and conformation transition for PS with *M*_n_ = 200,000 Da.

In the past few decades, due to the obstacles of synthetic technology, polymer chain conformation could only be analyzed by random labeling in FRET experiments. However, random labeling would result in an indeterminate distribution of fluorophores on the chain, and a randomly labeled polymer has an infinite number of chain contour lengths separating every two donor and acceptor pairs so that a distribution of rate constants is obtained that greatly complicates the quantitative analysis of the fluorescence data, which can only help us to understand the conformation of polymer chain qualitatively [20]. Based on this consideration, we are eager to simplify the quantitative analysis of polymer chain conformation by site-specific labeling, so our group developed a series of site-specific labeling strategies, including chain-end labeling [30,31].

Compared with random labeling on chains, the strategy of chain-end labeling can ensure the defined distribution of fluorophores, which can reflect the polymer chain conformation more directly. Even if there exist failed chains, including unlabeled chains, polymer chains with both acceptors at two ends, polymer chains with an acceptor at one terminal, and polymer chains with a donor at one terminal, the poorly defined chains do not furnish any disadvantageous effects for quantitative analysis. Wilemski and Fixman demonstrated theoretically in the 1970s that a monodisperse short chain yields a single end-to-end cyclization rate constant, and the presence of these well-defined polymer chains helps to simplify the analysis of fluorescence experiments [45,46]. In this article, we are more inclined to make a comparison of the behavior between long randomly labeled and short monodisperse end-labeled chains. The fluorescence experiments of polymer chains with chain-end labeling were performed as follows.

**Figure 3:**
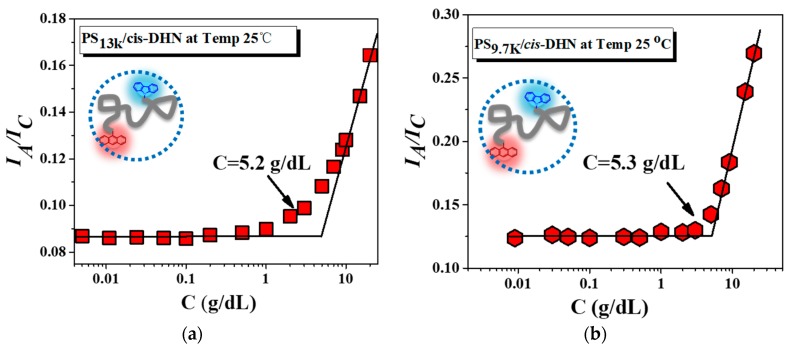
The *I_A_*/*I_C_* value as a function of concentration for (**a**) Cz-PS_13K_-An and PS and (**b**) An-PS_9.7K_-Cz and PS in DHN at 25 °C.

The fluorescence experiments were carried out on the PS samples with lower molecular weights (*M*_n_ = 13,000 Da and *M*_n_ = 9700 Da). When fluorophores are labeled at the chain terminals, the distance between donor and acceptor indicates the end-to-end distance, which directly reflects polymer coil size. Figure 3 shows *I_A_*/*I_C_* as a function of the concentration of PS_13k_ and PS_9.7k_. Two regions are also observed for dilute to semidilute solutions. Similarly, a break point at 5.2 g/dL for PS_13k_ and a break point at 5.3 g/dL for PS_9.7k_ can be observed. However, the values of *I_A_*/*I_C_* remain almost unchanged below the transition point, which is different from that of PS with high molecular weight (*M*_n_ = 200,000 Da), as mentioned above. That the value of *I_A_*/*I_C_* does not change much for PS_13k_ and PS_9.7k_ in a dilute solution is understandable because at the same concentration, shorter polymer coils are more separated compared with the long chains. According to the ratio of excimer to monomer fluorescence intensity, *I_E_*/*I_M_*, Qian found that *I_E_*/*I_M_* keeps consistent in dilute solutions. These data are in agreement with the results of Qian [22].

**Figure 4:**
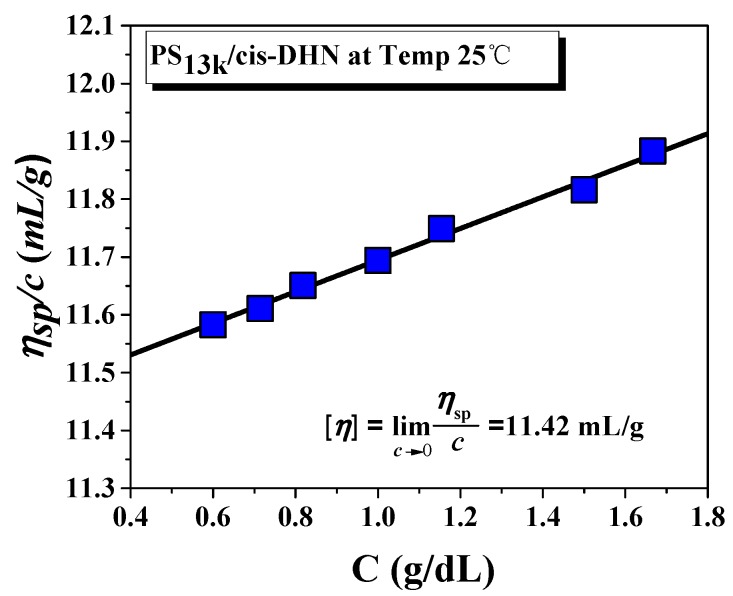
The reduced viscosity *η_sp_*/*c* as a function of concentration for PS (with *M*_n_ = 13,000 Da) in DHN at 25 °C.

To check the C* value of PS with a low molecular weight, a viscosity experiment of PS_13k_ solutions was also carried out. Figure 4 shows the reduced viscosity value *η_sp_*/*c* as a function of concentration for PS_13k_ in DHN at 25 °C. The intrinsic viscosity of PS_13K_/DHN solution is 11.42 mL/g. So the critical overlap concentration, taken as the inverse of intrinsic viscosity, is determined to be 8.7 g/dL [47], which is slightly larger than the break point from the fluorescence measurement. Such a mismatch is probably due to the influence of diffusion-enhanced FRET [48]. In solutions, the FRET signal comes from static and dynamic items [49]. Apparently, in our research, taking the impact of diffusion into account is essential. Makarow considered that donor–acceptor diffusion occurs in three dimensions and related an equation, $R_{\mathrm{rms}}={\left(6{D\tau }_{D}\right)}^{1/2}\;$ [50]. For PS with *M*_n_ = 200,000 Da, 0.4 units of **Cz** and 2.2 units of **An** exist on a signal chain, which results in a relatively large distance between **An** and **Cz** that makes the diffusion item negligible [24]. However, for PS with *M*_n_ = 13,000 Da, the diffusion-enhanced FRET becomes non-negligible. It is clear that the ends of a chain have a greater degree of freedom than units in the chain. The root-mean-square donor–acceptor displacement effected by diffusion during the excited lifetime of **Cz** is relatively large compared to the mean-square end-to-end distance, especially for the short chain. With the aid of diffusion, the FRET efficiency is enhanced, resulting in an earlier conformational transition at a lower concentration.

## 4. Conclusions

In this study, intrachain FRET was used to characterize the conformation evolution of polymer chains from dilute to semidilute solutions. Random and site-specific labeling strategies were utilized to prepare FRET-responsive PS with high molecular weight (*M*_n_ = 200,000 Da) and low molecular weight (*M*_n_ = 13,000 and 9700 Da), respectively. The behaviors between long randomly labeled and short monodisperse end-labeled chains were compared. According to the ratio of **An** and **Cz** intensities, *I_A_*/*I_C_*, the conformation of polymer chain can be inferred. It is clear that there is a conformational transition concentration based on fluorescence spectra. Above the concentration transition point, significant intrachain collapse occurs. Below the concentration transition point, slight conformational change of long polymer chains occurs in a dilute solution, whereas there is no significant conformational change for short polymer chains. The transition point matches C* determined from viscosity measurements for PS with high molecular weight but slightly larger than that for PS with low molecular weight because of the diffusion-enhanced FRET.

## Figures and Tables

**Table 1:** The parameters of PS samples.

Polymer	Mn	PDI	Mol % of Cz	Mol % of An
PS	2.05 × 10^5^	1.05	/	/
PS-An-Cz	2.05 × 10^5^	1.05	0.0214	0.114
PS	12,500	1.09	/	/
An-PS-Cz	13,300	1.10	0.573	0.515
PS	9800	1.10	/	/
An-PS-Cz	9700	1.09	0.622	0.300
